# 4138 Development of an Antibiofilm Resorbable Membrane for Treating Peri-implantitis

**DOI:** 10.1017/cts.2020.364

**Published:** 2020-07-29

**Authors:** Zhou Ye, Joseph R. Rahimi, Nicholas G. Fischer, Hooi Pin Chew, Conrado Aparicio

**Affiliations:** 1 University of Minnesota CTSI; 2 University of Minnesota

## Abstract

OBJECTIVES/GOALS: Peri-implantitis is the inflammation of peri-implant mucosa and subsequent loss of supporting bone. Its treatment is only <40% successful mainly due to persistent bacterial infection. The goal of this project is to increase success rates by developing a robust antibiofilm multi-biomolecular membrane that can be placed around implant surfaces. METHODS/STUDY POPULATION: A collagen membrane was soaked in the antimicrobial peptide GL13K solution overnight to form an interpenetrating fibrillary network. The nanostructure of the membrane was imaged with scanning electron microscope (SEM). The hydrophobicity of the membrane was analyzed by water contact angle (WCA) measurements. The biodegradability was tested in a 0.01 mg/mL Type I collagenase solution for up to 5 weeks. The antimicrobial activity of the membrane was assessed with Gram-positive oral bacteria Streptococcus gordonii. The cytotoxicity was evaluated by culturing human gingival fibroblasts (HGF), and the osteogenesis was assessed using preosteoblasts MC3T3. Pure collagen membrane was used as the control. Statistical significance (p<0.05) was determined by one-way ANOVA with Tukey’s HSD test. RESULTS/ANTICIPATED RESULTS: The antimicrobial peptide GL13K self-assembled to short fibrils (< 1 µm long), which entangled with the larger collagen fibers (around 200 nm in diameter). The collagen fibers presented characteristic periodic banding structures, which provided biomimetic cues for cell behavior as extracellular matrix. The interpenetrated GL13K fibrils turned the highly hydrophilic collagen membrane to a hydrophobic membrane (WCA = 135 °) and significantly reduced the rate of degradation by collagenases. The developed membrane was efficient in preventing the attachment of *S. gordonii*. A large portion of the attached bacteria was killed on the surface of the membrane. The incorporation of GL13K did not affect the cytocompatibility of the membrane for HGF. DISCUSSION/SIGNIFICANCE OF IMPACT: We developed an antibiofilm membrane with interpenetrating collagen and antimicrobial peptide fibrils. The strong antimicrobial activity and low cytotoxicity support its further translational evaluation as scaffolds for increasing success rate in treating peri-implantitis.

